# Comprehensive Enhancement in Thermomechanical Performance of Melt-Extruded PEEK Filaments by Graphene Incorporation

**DOI:** 10.3390/polym13091425

**Published:** 2021-04-28

**Authors:** Srinivasarao Yaragalla, Muhammad Zahid, Jaya Kumar Panda, Nikolaos Tsagarakis, Roberto Cingolani, Athanassia Athanassiou

**Affiliations:** 1 Smart Materials, Istituto Italiano di Tecnologia, Via Morego 30, 16163 Genova, Italy; 2 Graphene Labs, Istituto Italiano di Tecnologia, Via Morego 30, 16163 Genova, Italy; jayakumar.panda@iit.it; 3 Humanoids and Human Centered Mechatronics, Istituto Italiano di Tecnologia, Via Morego 30, 16163 Genova, Italy; Nikolaos.Tsagarakis@iit.it; 4 Istituto Italiano di Tecnologia, Via Morego 30, 16163 Genova, Italy; Roberto.Cingolani@iit.it

**Keywords:** graphene nanoplatelets, melt-extrusion, polymer nanocomposites, PEEK, storage modulus, thermal stability

## Abstract

A simple and scalable fabrication process of graphene nanoplatelets (GnPs)-reinforced polyether ether ketone (PEEK) filaments with enhanced mechanical and thermal performance was successfully demonstrated in this work. The developed PEEK–GnP nanocomposite filaments by a melt-extrusion process showed excellent improvement in storage modulus at 30 °C (61%), and significant enhancement in tensile strength (34%), Young’s modulus (25%), and elongation at break (37%) when GnP content of 1.0 wt.% was used for the neat PEEK. Moreover, the GnPs addition to the PEEK enhanced the thermal stability of the polymer matrix. Improvement in mechanical and thermal properties was attributed to the improved dispersion of GnP inside PEEK, which could form a stronger/robust interface through hydrogen bonding and π–π* interactions. The obtained mechanical properties were also correlated to the mechanical reinforcement models of Guth and Halpin–Tsai. The GnP layers could form agglomerates as the GnP content increases (>1 wt.%), which would decline neat PEEK’s crystallinity and serve as stress concentration sites inside the composite, leading to a deterioration of the mechanical performance. The results demonstrate that the developed PEEK–GnP nanocomposites can be used in highly demanding engineering sectors like 3D printing of aerospace and automotive parts and structural components of humanoid robots and biomedical devices.

## 1. Introduction

Polyether ether ketone (PEEK) is a high-performance, robust, semi-crystalline, and chemically inert thermoplastic polymer [1,2], with high impact damage resistance comparable to epoxy resins [1,3], which maintains its mechanical properties even at high temperatures ~300 °C [4,5]. Therefore, PEEK-based components are found in demanding engineering applications, such as piston parts, bearings [6], aircrafts structures [7], biomedical implants [8], to name a few. In the past, the virgin PEEK polymer has been frequently reinforced with carbon-based nanofillers, such as carbon nanotubes (CNTs) and carbon nanofibers (CNFs), to enhance its mechanical [9,10], tribological [6,11], thermal [12,13], and electrical properties [14,15] and displayed substantial improvements. However, these nanomaterials (CNFs and CNTs) are expensive (e.g., multiwall CNTs cost 50 to 100 $/gram) and add a high value to the final composites [16]. In contrast, graphene’s role (another carbon-based nanofiller [17]) in the PEEK reinforcement is rarely mentioned in the literature [6,18,19,20]. Graphene, with its unprecedented mechanical (Young’s modulus can reach 1 TPa) [21], thermal (3080–5150 W/m K) [22] and electrical (order of 10^8^ s/m) [23] properties, has outperformed its fillers counterparts (metallic/ceramic nanofillers) in other polymer composites [24]. Therefore, it is worth investigating the reinforcement of PEEK using graphene fillers to develop high-performance composites.

Previously, Tewatia et al. [18] disclosed dynamic thermomechanical properties of graphene (bi/tri-layers)-filled PEEK nanocomposites prepared by melt mixing. The authors have reported a ~29% improvement in the storage modulus at 5.0 wt.% of graphene loading. However, the tensile strength and Young’s modulus of the prepared nanocomposites did not show any noteworthy improvements. In another study, Puértolas et al. [6] have fabricated multilayer graphene (≥ 8 layers)-based PEEK nanocomposites using melt-blending and injection molding techniques. The authors have studied the effect of filler concentration (1.0–10.0 wt.%) on the prepared nanocomposites’ tribological and mechanical properties. The graphene-filled PEEK nanocomposites demonstrated ~2% and ~44% improvement in Young’s modulus for 1 wt.% and 10 wt.% of graphene, respectively. On the other hand, they have noticed a decline in ultimate tensile strength and elongation at break upon graphene loading. Apart from pristine graphene, graphene oxide (GO) has been studied for PEEK reinforcement. For instance, in a recent work, He et al. studied mechanical properties of the GO-integrated PEEK nanocomposites [20]. The GO-based PEEK nanocomposites were prepared by a twin-screw extruder using 0.1 to 5.0 wt.% filler contents and at 2.0 wt.% concentration demonstrated ~4% and ~34% improvements in Young’s modulus and maximum elongation at break, respectively. Further, an increase in the GO content up to 5.0 wt.% could not produce any significant improvement, although the maximum elongation at break was reduced by ~11%. The authors have attributed this degradation to the agglomeration of the GO fillers at higher concentrations. Hwang et al. [25] also studied the incorporation of carboxylated multiwall carbon nanotubes (MWCNTs) together with GO in the PEEK. The storage modulus of the hot-pressed MWCNTs/GO-PEEK hybrid nanocomposites showed significant improvement in the thermomechanical properties. The storage modulus reached ~3.3 GPa from ~2.2 GPa, corresponding to ~50% enhancement. The so far reported works indicate that GO is advantageous for its composites’ mechanical properties compared to pristine graphene [26]. However, the known methods to prepare the GO include strong chemicals (nitric or sulfur acids and potassium chlorate) [27] and produce noxious emissions [28]. In contrast, the multilayer graphene or graphene nanoplatelets (GnPs, >8 carbon layers) [29] can be directly prepared by liquid-phase exfoliation (in organic solvents, ionic liquids or water/surfactant) in large quantities [30,31], are inexpensive (<2 $/gram) [32,33], and therefore, would be ideal fillers for the improvement of mechanical and thermal properties of PEEK nanocomposites.

In this work, an optimized reinforcement of PEEK polymer with GnPs, using the GnP fillers’ physical adsorption onto PEEK polymer followed by a melt-extrusion process, is reported. The extruded nanocomposite filaments were studied for their mechanical properties concerning the filler concentration. Mechanical properties of the developed nanocomposites were compared to theoretical models to better explain the behavior of the prepared PEEK–GnP system. Furthermore, the effect of GnP loading on the PEEK polymer chain mobility/confinement is discussed in detail concerning the thermal properties of the prepared nanocomposites. The improvement of all the mechanical and thermal properties of the optimized nanocomposites for the neat PEEK indicates that they can be used in highly demanding engineering sectors, i.e., robotics, constructions, aviation, etc., by developing durable structural parts using common technologies like injection molding or 3D printing.

## 2. Materials and Methods

### 2.1. Materials

GnP powder (grade- Pure G^+^) was kindly provided by Directa plus, Milan, Italy. For detailed characterization of the GnP powder, refer to [34]. Briefly, the GnP fillers are composed of >8 atomic layers, have an average lateral size of ~600 nm and an aspect ratio (*Ø_f_*, ratio of lateral size over thickness) of ~75. PEEK (Larpeek 50) granules were obtained from Ultra Polymers, Milan, Italy. Ethanol was received from Sigma-Aldrich, St. Louis, MO, USA, and was used without further purification.

### 2.2. Fabrication of PEEK–GnP Nanocomposites

#### 2.2.1. Adsorption of GnPs on the Surface of PEEK

GnP powder (0.5, 1.0 and 3.0 g) was initially dispersed in 200 mL ethanol with the help of a bath sonicator (at 59 kHz, 135 W, and amplitude of 100%, Savatec-Torino, Italy) for 2 h. PEEK polymer pellets (diameter ~3 mm) were ground to powder form (grain size ~1 mm) using an IKA Pilotina dry mill (IKA, Königswinter, Germany). Next, the PEEK powder (99.5, 99.0 and 97.0 g) was added to the beaker containing GnPs dispersion (200 mL) in ethanol and stirred for 24 h using a mechanical mixer rotated at 200 rpm until the mixture was semi-dry. Then, before extrusion, the whole mixture was further dried in a convection oven at 60 °C for 48 h to eliminate the trapped solvent. Using this procedure, the GnP flakes were physically deposited or adsorbed onto the PEEK powder resulting in a core–shell structure (see an illustration of the fabrication process in Supplementary Figure S1). The neat PEEK powder was also treated in the same way. The percentages of the PEEK and GnP fillers in the prepared compositions are listed in Table 1.

#### 2.2.2. Extrusion of Filaments of the PEEK–GnP Nanocomposites

The prepared PEEK powder (grain size ~1 mm) with or without deposited GnP fillers was extruded by melt compounding in a co-rotating double-screw extruder (2C15-45 L/D, *Luigi Bandera*-Busto Arsizio, Italy) using a temperature profile along the barrel from 380 to 400 °C, screws rotating at ~100 rpm and outlet channel size 3.30 mm. The extruded PEEK and PEEK–GnP nanocomposites (hereafter, also filaments), with diameter ~1.05 ± 0.04 mm, were cooled down at room temperature (see Supplementary Figure S1). The extruded filament draw ratio (filament diameter at outlet channel/filament diameter collected) was ~3.14 ± 0.04 [35].

### 2.3. Characterization

Morphological analysis of the neat PEEK and PEEK–GnP filaments was done using scanning electron microscopy (SEM, JEOL-6490AL, Tokyo, Japan). The filaments were cryo-fractured using liquid nitrogen before the SEM analysis to preserve the structure of the nanocomposite.

Attenuated total reflectance Fourier-transform infrared (ATR-FTIR) spectra of the prepared composites were recorded with a Bruker Vertex 70v (Bruker, Billerica, MA, USA) using wavenumber ranging from 1000 to 4000 cm^−1^ with a resolution of 2 cm^−1^, averaging 128 scans. Five spectra of each composition were collected.

The chemical composition of the GnP powder (grade Pure G^+^, DirectaPlus, London, UK) and chemical interactions associated with GnP and PEEK was analyzed using X-ray photoelectron spectroscopy (XPS) having an electron spectrometer (Lab2, Specs, Berlin, Germany) equipped with a monochromatic X-ray source (set at 1486 eV) and with a hemispherical energy analyzer (Phoibos, HSA3500, also from Specs, Berlin, Germany). The applied voltage of the Al Kα X-ray source was set at 13 kV and the applied current at 8 mA. The pressure in the analysis chamber was ≈1 × 10^−9^ millibar. The large area lens mode was employed for both wide and narrow scans. For the wide scan, the energy pass was 90 eV, and the energy step was 1 eV. For the narrow high-resolution scan, the energy pass was 30 eV, and the energy step was 0.1 eV. A flood gun was used to neutralize the surface charge, having an energy of 7 eV and a filament current of 2.2 A.

Room temperature Raman measurements were conducted using a Renishaw InVia micro-Raman spectrometer (Wotton-under-Edge, UK )with a 50 × objective (numerical aperture of 0.75) with an excitation wavelength of 785 nm line of a diode laser. A very low incident power (less than 0.5 mW) was used to avoid sample damage. The scattered light was detected in a back-scattering geometry dispersed by 1200 grooves mm^−1^ grating.

X-ray diffraction (XRD) analysis was conducted on a PANalytical Empyrean X-ray diffractometer (Malvern, UK) equipped with a 1.8 kW CuKα ceramic X-ray tube, PIXcel3D 2 × 2 area detector and operating at 45 kV and 40 mA. The X-ray diffraction patterns were recorded in the air at room temperature using a zero diffraction quartz substrate, Parallel-Beam (PB) geometry and symmetric reflection mode.

The thermal stability of the as-prepared PEEK and of its GnPs-filled nanocomposites was also evaluated by the Thermogravimetric Analysis TGA Q500 system (TA instruments, New Castle, DE, USA). Each measurement was done on an 11 mg sample weighed in an aluminum pan and heated from 30 to 800 °C with a heating rate of 10 °C/min under a nitrogen environment. The gas flow rate was maintained at 50 mL/min. Additionally, the fabricated filaments’ thermal behavior was investigated through differential scanning calorimetry DSC, S2-00579 (TA instruments, New Castle, DE, USA). The samples of 20 ± 1 mg of neat PEEK and PEEK–GnP nanocomposites were weighed in a sealed aluminum pan and heated from 20 °C to 400 °C with a rate of 10 °C/min under nitrogen environment. After equilibrating at 400 °C for one minute, the samples were cooled down from 400 °C to 20 °C with a rate of 10 °C/min. Again, after equilibrated at 20 °C for one minute, the samples were further subjected to a second heating cycle with the same parameters concerning the reference empty aluminum pan. The calibration of the instrument was done using Indium before the analysis.

The prepared PEEK–GnP filaments’ tensile properties were investigated with an Instron dual column tabletop universal testing system 3365 (Norwood, MA, USA) at a 10 mm/min strain rate. The engineering stress–strain measurements were conducted on five different specimens for each sample. Dynamic mechanical analysis (DMA) measurements were carried out using DMA Q800 V21.2 Build 88 (TA instruments, New Castle, DE, USA)with fiber clamps under tension mode, using the temperature ramp method. The samples (length 12 mm and diameter ~1 mm) were heated from 30 °C to 195 °C, and frequency was maintained at 10 Hz for all the samples.

## 3. Results and Discussion

### 3.1. Morphology Observation

**Figure 1:**
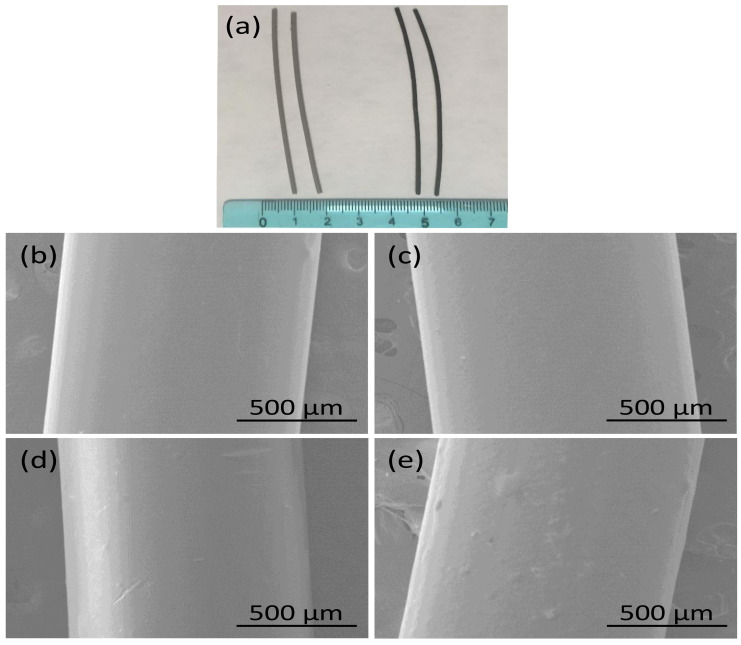
(**a**) Photographs of extruded neat PEEK (left) and GnP-loaded PEEK filaments (right). Surface SEM images of (**b**) Neat PEEK (**c**) 0.5 wt.% (**d**) 1.0 wt.% (**e**) 3.0 wt.% GnP fillers-loaded PEEK filaments.

**Figure 2:**
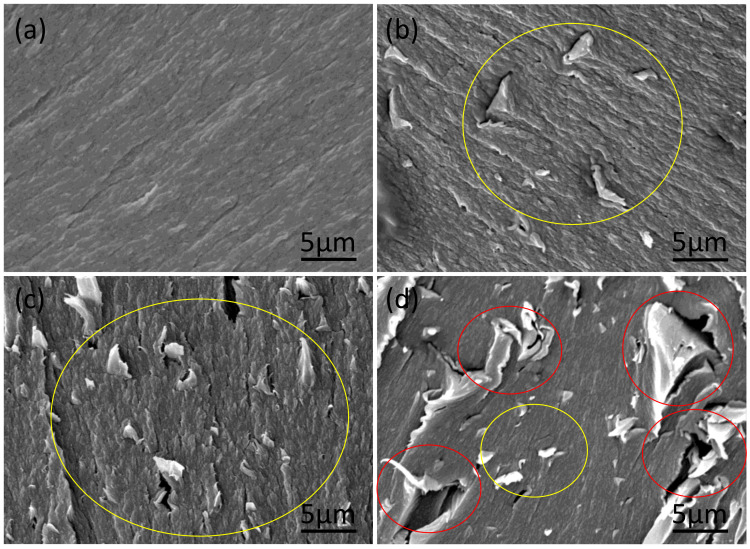
Tensile fractured cross-sectional SEM images of (**a**) Neat PEEK and PEEK loaded with (**b**) 0.5 wt.% (**c**) 1.0 wt.% (**d**) 3.0 wt.% of GnP fillers.

Photographs and SEM images of the developed PEEK filaments with and without GnP fillers are displayed in Figure 1. The PEEK and PEEK–GnP filaments, shown in Figure 1a, appear smooth, having a diameter ~1.05 mm ± 0.045 mm. Their corresponding SEM images are shown in Figure 1b–e. It is clear from SEM images that all the filaments exhibit smooth surfaces without voids or other defects. However, in the electron microscope, it can be seen that the surface roughness slightly increases for the composite at 3 wt.% of GnPs loading. The cross-section morphology of tensile fracture filaments was analyzed (see Figure 2) to investigate the fracture mechanism. The filaments at lower loadings (0.5 & 1 wt.% GnP) show drawn morphology and improved GnP dispersion (yellow circles, see Figure 2b,c) with ductile fracture (higher elongation), indicating a high degree of plastic deformation, whereas at higher loading at 3 wt.% exhibited aggregated GnP structures with voids (red circles, see Figure 2d) similar to brittle fracture materials. Similar morphology behavior has also been reported by He et al. [20] and Pascual et al. [36]. This analysis is well correlated with the obtained mechanical properties, as is conferred in the subsequent section. Moreover, cryo fractured surfaces were also analyzed to understand the dispersion of GnP inside PEEK filaments deeply. The cross-section SEM image of the extruded neat PEEK polymer also appears curvy waveforms with some irregularities due to its brittleness at lower (cryogenic) temperatures. (See Supplementary Figure S2a). In contrast, cross-section SEM images of the PEEK filaments incorporating GnP fillers at different concentrations reveal the presence of graphene layers and voids, indicated with red and yellow arrows, respectively (see Supplementary Figure S2b–d). The voids increase in frequency and size with increasing filler concentration. They are most likely created as the GnPs, aggregated or not, are pulled away from the polymer matrix during the cryo-fracturing process. One can easily notice the improved GnPs dispersion inside the PEEK at lower loadings, such as 0.5 wt.% and 1.0 wt.% (see Supplementary Figure S2b,c) corresponding to samples PEEK–GnP0.5 and PEEK–GnP1.0, respectively. The improved dispersion of graphene layers at these concentrations on the cryo-fractured surfaces also indicates a strong filler–polymer adhesion, as the fillers were rarely detached during the fracturing process leaving very few and difficult to find voids. On the other hand, the sample PEEK–GnP3.0 was filled with the highest GnPs concentration, e.g., 3.0 wt.% shows aggregated nanofillers and a substantial increase in the void formation and the voids’ size (see yellow arrows in Supplementary Figure S2d). This morphology analysis is somewhat different from the findings of Puértolas et al. [6], where they adopted different sample preparation methods.

### 3.2. Characteristics of Molecular Structure

**Figure 3:**
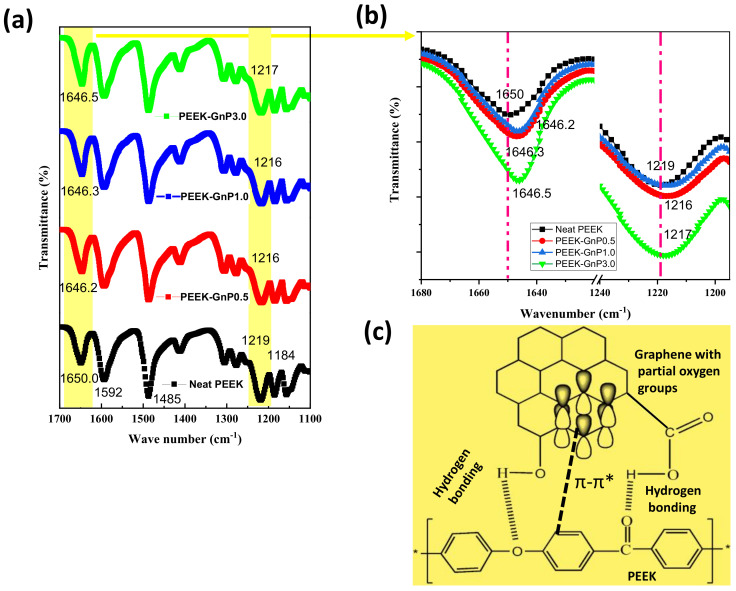
(**a**) ATR-FTIR spectra of the neat PEEK and PEEK–GnP nanocomposites with different filler contents. The identification of the PEEK footprints is included. (**b**) FTIR stretching modes of carbonyl (-C=O) and ether (C–O–C) groups before and after GnPs loading. (**c**) A schematic representation of the hydrogen bonding and π–π* interactions between graphene and PEEK polymer.

The developed PEEK–GnP nanocomposites were studied by ATR-FTIR and XPS to understand the possible chemical interactions between the GnP fillers and the PEEK matrix. Figure 3a presents ATR-FTIR spectra of the neat PEEK and PEEK–GnP nanocomposites. The neat PEEK displayed characteristic peaks at 1650 cm^−1^, 1592 cm^−1^, and 1485 cm^−1^ corresponding to the carbonyl, aromatic C–C, and C–H vibrations, respectively [37]. Moreover, the peaks obtained at 1219 cm^−1^ and 1184 cm^−1^ are due to the symmetric and asymmetric stretching vibrations of C–O–C linkages in the PEEK structure, respectively [38,39]. In the case of PEEK–GnP nanocomposites, -C=O and C–O–C peak positions of the PEEK (highlighted with yellow color) are shifted towards lower wavenumbers ~4 cm^−1^ and ~2–3 cm^−1^, respectively, as seen in Figure 3b, which indicates the existence of hydrogen bonding (H-bonding) interactions among the -C=O and -C–O–C groups of the PEEK polymer and the oxygenated moieties of GnPs. XPS (X-ray photoelectron spectroscopy) analysis of the pure GnP powder shows the presence of oxygenated groups (-O–H and –CO-O–H, 6.63%) in the GnPs (see Supplementary Figure S3a) that can interact with the PEEK polymer. These interactions are more visible for the carbonyl and ether peaks of the PEEK polymer, as seen in Figure 3b, and they are in good agreement with previous reports [10,20,40,41]. The chemical composition of PEEK–GnP1.0 is illustrated by XPS spectra (see Supplementary Figure S3b). The peaks at 284.82 eV, 286.10 eV, and 289.02 eV correspond to C–C/C–H, C–O, and C=O chemical groups [20]. The additional peak appeared at 291.53 eV is attributed to the π–π* interaction (see Supplementary Figure S3b), suggesting that formation π–π* conjugated chemical structure and strong chemical interaction between PEEK and GnP, it is well-known that, while π–π* bond (291.53 eV) is absent in neat PEEK [20]. Similarly, chemical interactions (hydrogen bonding and π–π*) were identified among carbon-based fillers and PEEK composites in the past [10,20]. The chemical interactions observed here (through FT-IR and XPS) are also validated by a significant improvement in the mechanical properties, as is discussed next.

**Figure 4:**
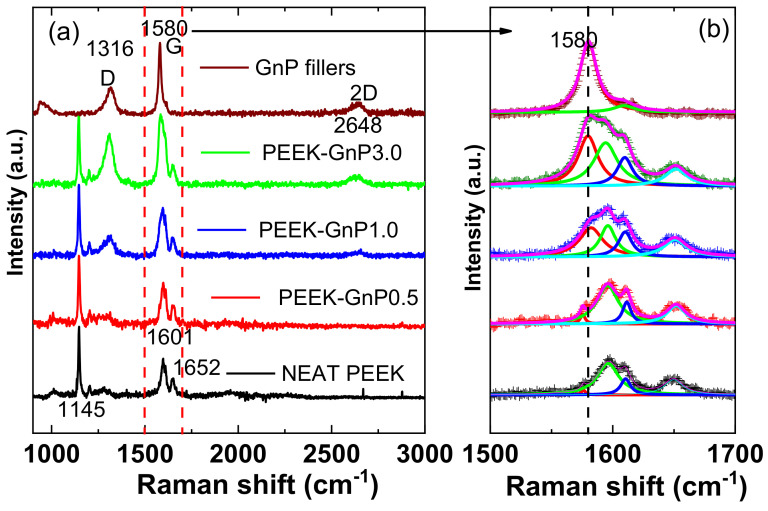
(**a**) Raman spectra of the neat PEEK and PEEK–GnP nanocomposites and (**b**) deconvolution of the overlapped G band of the GnPs and -C=C stretching vibration of the PEEK structure. The red peak is associated with the graphitic G band.

Raman characterization of the neat PEEK and PEEK–GnP nanocomposites was also performed to further investigate the graphene–polymer interactions. Figure 4 shows normalized Raman spectra of the neat PEEK and PEEK–GnP nanocomposites. The neat PEEK’s Raman spectrum shows peaks at 1145 cm^−1^, 1601 cm^−1,^ and 1652 cm^−1^ corresponding to the C–O, C=C, and C=O stretching vibrations, respectively [42,43]. The coupling peaks of the neat PEEK that appeared at 1601 cm^−1^ and 1652 cm^−1^ are further associated with polymer crystallinity [6]. On the other hand, the pristine GnP powder (as-received) shows characteristic peaks at 1316 cm^−1^, 1580 cm^−1,^ and 2648 cm^−1,^ representing the formation of D band (disordered structure of graphene), graphitic G band (C–C stretching in sp^2^ hybridized carbon atoms) and 2D band (the second-order of zone-boundary phonons), respectively, whereas the position of the 2D band at 2648 cm^−1^ indicates that GnP contains ≥8 layers [44,45,46]. As the graphene loading increases, one can notice that the intensity of D and 2D bands increases in the Raman spectra, indicating the successive loading of the GnPs into the composites. Since the G band signal of PEEK–GnP nanocomposites overlaps with the neat PEEK signal, as seen in Figure 4a, the overlapped peaks have been deconvoluted in Figure 4b. The deconvoluted G bands reveal the increasing contribution of the G band (red color) with the addition of the GnP fillers, indicating the formation of more sp^2^ conjugated carbons inside the PEEK polymer.

**Figure 5:**
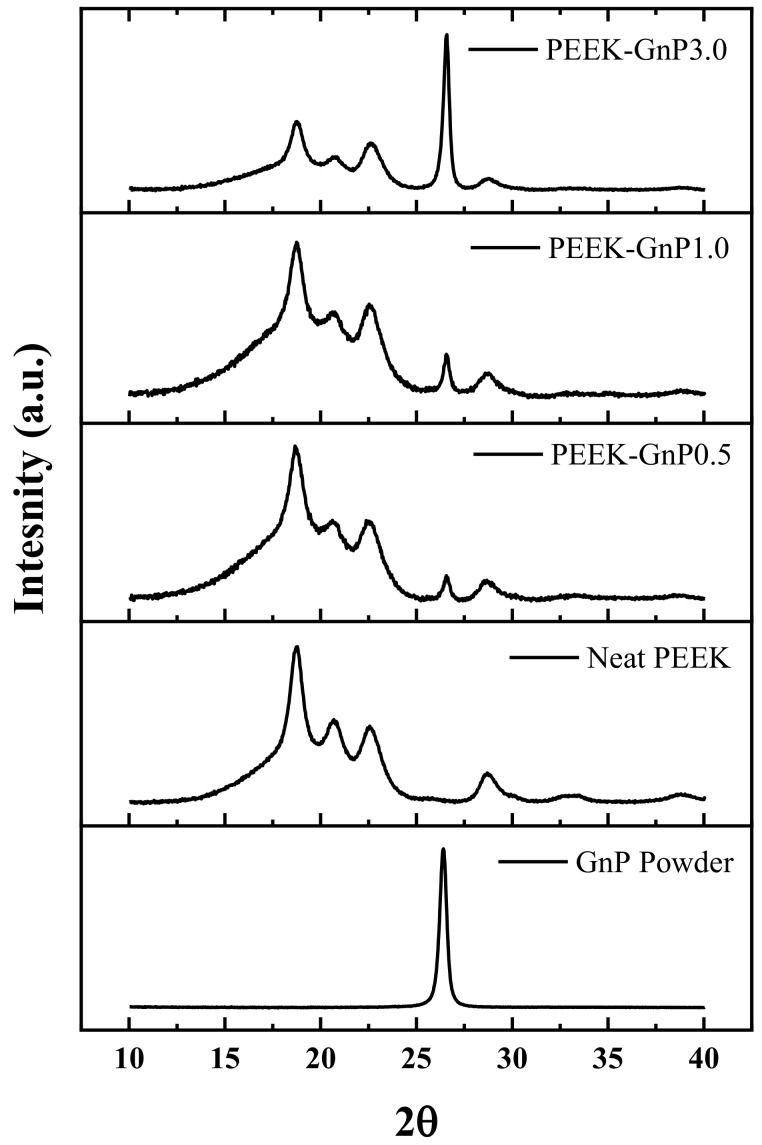
XRD patterns of the neat PEEK and GnP-filled PEEK nanocomposites.

In addition to ATR-FTIR and Raman analysis, an XRD study was also carried out to understand the nanocomposites’ microcrystalline structure. The XRD data of the nanocomposites are shown in Figure 5. The neat GnP powder exhibits a sharp peak at 2θ ≈ 27°, indicating the 002 planes of the crystalline hexagonal six-membered graphitic structure [47]. On the other hand, the neat PEEK exhibits XRD reflections at 2θ ≈ 18.7°, 20.7°, 22.6°, and 28.7° corresponding to the 110, 111, 200, and 211 planes of the orthorhombic PEEK structure, respectively [6]. Scherrer equation [48] was used to estimate average crystallite size (L) in the neat PEEK and PEEK–GnP nanocomposites (see details in Supplementary Materials, Section SI-4). The XRD reflections at 110, 111, and 200 planes were used to estimate the average crystallite size and are reported in Table S1 (see Supplementary Materials). The crystallite size change is lesser than 4.1%, with the lower GnP loadings at 0.5 & 1 wt.%. In contrast, at higher loading (3 wt.% GnP), the average crystallite size (5.63 nm) was reduced to 12.4% than that of the neat PEEK (6.43 nm), which would be one of the factors for its poor tensile strength and lower elongation (refer Section 3.4). Moreover, relative intensities of peak (110) significantly changed for 3 wt.% GnP-loaded sample compared with neat PEEK and at lower loadings (0.5 and 1wt.%). It may be concomitant with the orientation of PEEK crystals induced by the high concentration of GnP filler and is also associated with transcrystallanity on GnP surface, which is minimal at lower loadings (0.5&1 wt.%) of GnP. Similarly, Puértolas and co-authors have also reported a decline in crystallite size of 12.8% of the PEEK polymer at 3 wt.% of graphene loading [6] where they noticed lower tensile properties (tensile strength &elongation at break).

### 3.3. Thermal Analyses

**Figure 6:**
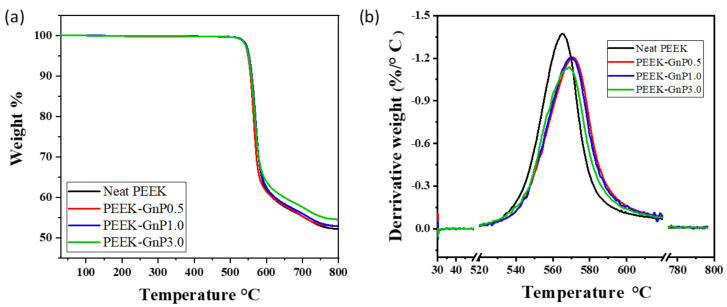
Thermogravimetric analysis, (**a**) TGA and (**b**) DTGA, of the neat PEEK and PEEK filled with 0.5, 1.0, and 3.0 wt.% of GnPs.

Thermogravimetric analysis (TGA) of the neat PEEK and PEEK–GnP nanocomposites is summarized in Figure 6. It is evident from Figure 6 that the addition of the GnPs to the PEEK matrix enhances its thermal stability. In particular, in Figure 6b, the first derivative of the TGA (DTGA) curves reveals that thermal decomposition temperatures of the PEEK–GnP nanocomposites (568 to 572 °C) are higher compared to the neat PEEK (565 °C). The increase in the degradation temperature for the PEEK–GnP nanocomposites further validates the chemical interactions (H-bonding & π–π*) associated with the PEEK and GnPs. Graphene has already been proven as a thermal stabilizer in many polymers due to its impermeable structure to gases produced during thermal decomposition [49]. In particular, graphene can create tortuous gas diffusion pathways, thanks to its platelet geometry [50]. In the PEEK case, the common byproducts during thermal decomposition are CO, CO_2_, phenols, and aromatic ethers [51]. The incorporated GnPs can hinder the escape of such byproducts from the polymer matrix, improving its thermal stability. In addition, the GnPs may prevent the decomposition of ketone groups via molecular association, further stabilizing the polymer structure [18].

DSC analysis was performed to understand the effect of GnP on the microcrystalline structure of PEEK. The second heating melting DSC curves of the neat PEEK and PEEK–GnP nanocomposites with 0.5, 1.0, and 3.0 wt.% of GnP fillers are presented in supporting information (See Supplementary Figure S4). The melting temperature of the neat PEEK (~337.4 °C) also does not alter much with the GnPs loading. This further clarifies that the PEEK polymer’s core structure using polymer chain confinement has not been affected. The degree of crystallinity Xc (%) in the PEEK polymer was also estimated before and after GnPs inclusion using the following Equation (1) [52]:

(1) $$X_{c}\;=\;\frac{\Delta H_{m}}{\left(1-\phi \right)\Delta H_{0}}$$

where Δ*H*_0_ is the heat of fusion of the 100% crystalline PEEK taken as 130 J/g, *ϕ* is the weight fraction of the GnP fillers to the polymer, and Δ*H_m_* is the heat of melting estimated from integrating the melting peak for all the samples. The degree of crystallinity (*X_c_*) of the different compositions is listed in Table 2. The degree of crystallinity of the neat PEEK slightly lowers (~5.0%) with the addition of lower loading of the GnPs (0.5 and 1.0 wt.%). In contrast, at higher loading 3 wt.% GnP, ~10% decrease in crystallinity was noticed, which is one of the reasons for its observed low mechanical performance (refer Section 3.4). These results are in good agreement with the XRD analysis. Similarly, Puértolas and his coworkers have reported a reduction in the PEEK polymer’s crystallinity when graphene is incorporated [6]. In another report, Pascual et al. had observed that the slight decrease in crystallinity of PEEK (less than 5%) when reinforced with carbon nanotubes at lower loading (1 wt.%) could not decline its mechanical performance [53].

### 3.4. Thermomechanical Properties

**Figure 7:**
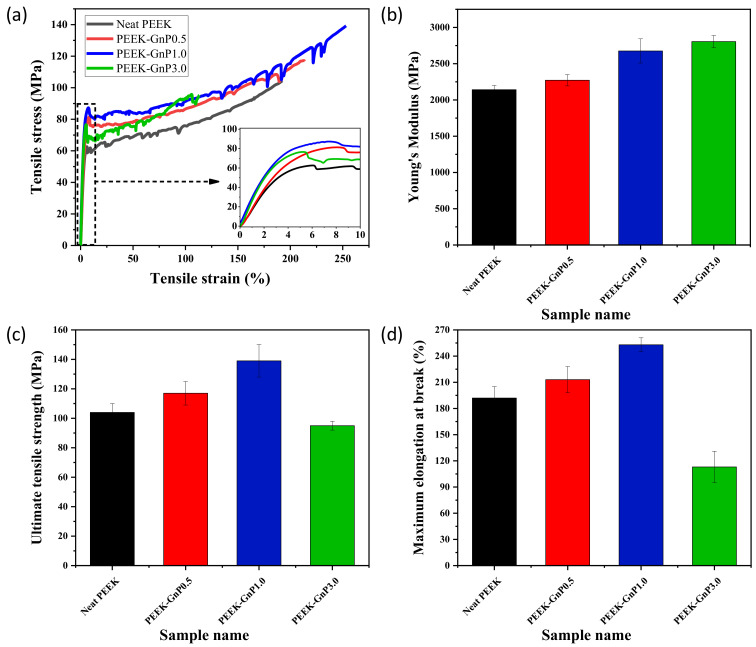
Tensile properties of the neat PEEK and PEEK–GnP nanocomposites filled at 0.5, 1.0, and 3.0 wt.% GnP content, (**a**) engineering stress–strain curves, (**b**) Young’s modulus, (**c**) ultimate tensile strength, and (**d**) maximum elongation at break.

Stress–strain curves and corresponding Young’s modulus, ultimate tensile strength, and elongation at break of the extruded neat PEEK and PEEK–GnP filaments are illustrated in Figure 7. In general, tensile properties (tensile strength and modulus) of the polymer composites are influenced by graphene fillers [25,54], and, as seen in Figure 7a, the stress–strain curves of all the composite samples appear improved with respect to the neat PEEK. In particular, Young’s modulus, ultimate tensile strength, and maximum elongation at break increased from 2142 ± 58 MPa, 104 ± 6 MPa, and 192 ± 13% (for the neat PEEK) to 2272 ± 78 MPa, 117 ± 8 MPa, and 213 ± 15%, respectively, for the nanocomposite with 0.5 wt.% concentration of GnPs (sample PEEK–GnP0.5), as shown in Figure 7b–d. Further addition of the GnPs to the PEEK polymer, arriving at 1.0 wt.% (sample PEEK–GnP1.0), produced substantial improvements in mechanical properties. For instance, Young’s modulus and ultimate tensile strength of the sample PEEK–GnP1.0 reached 2676 ± 166 MPa and 139 ± 11 MPa, corresponding to ~25% and ~34% improvements (compared to the neat PEEK), as shown in Figure 7b,c, respectively. Similarly, the maximum elongation at break reached 253 ± 8% (from 192 ± 13% for the neat PEEK), as shown in Figure 7d. This is due to the improved dispersion of the GnP fillers inside the PEEK matrix and the formation of a strong interface between fillers and matrix through hydrogen bonding and π–π* interactions. These factors contribute to optimizing the stress transfer from the PEEK matrix to the graphene fillers, inducing a strong reinforcing action of the GnP fillers [55,56,57]. The improvement in elongation at break (31%) and tensile strength (34%) at 1 wt.% of GnP loading demonstrated significantly better results than the previous reports [6,58,59]. In contrast, the samples PEEK–GnP3.0 with higher filler loading, i.e., 3.0 wt.% of GnPs, displayed ~41% and ~9% decrease in the maximum elongation at break and ultimate tensile strength, respectively. Nevertheless, Young’s modulus continued to increase, and it was improved by 31%, reaching 2805 ± 83 MPa (see Figure 7b–d). This can be associated with the enhanced aggregates of the GnP fillers that might have acted as stress concentration sites (see Supplementary Figure S2d), resulting in a decline in the mechanical performance. Now it is well established in the literature that at filler concentration > 1 wt.%, graphene agglomeration is unavoidable [57,60]. Summarizing, the extruded filaments of PEEK with 1.0 wt.% of GnPs concentration (PEEK–GnP1.0) presented the best mechanical performance compared to the other two nanocomposite samples that are PEEK–GnP0.5 and PEEK–GnP3.0.

**Figure 8:**
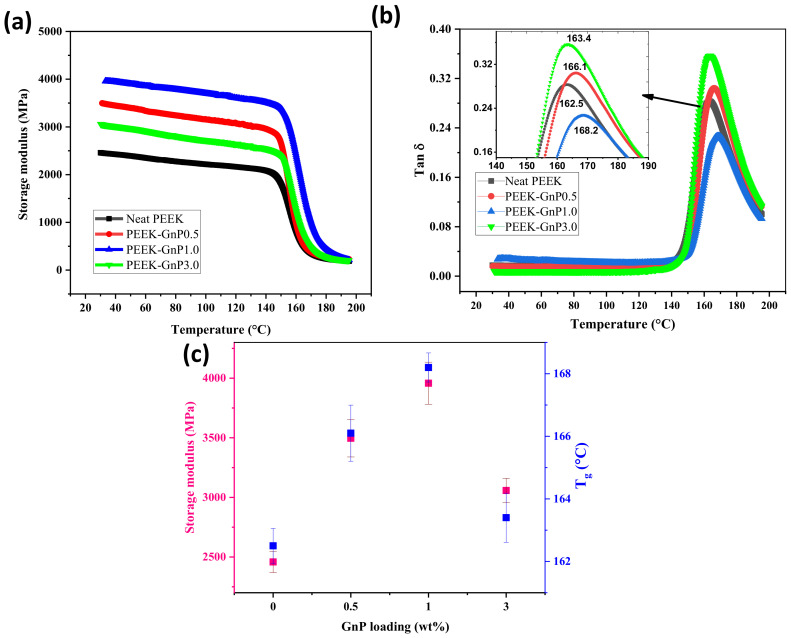
DMA properties of the neat PEEK and PEEK–GnP nanocomposites, (**a**) storage modulus vs. temperature, (**b**) tan δ curves and (**c**) storage modulus at 30 °C and glass transition temperature (*T*_g_) with respect to GnP loading.

Furthermore, the effect of concentration of the GnP fillers on the PEEK polymer’s storage modulus was also evaluated using DMA and is shown in Figure 8. In general, a change in any polymer’s storage modulus is associated with its rigidity, which ultimately reflects the material’s strength. Herein, the storage modulus of the PEEK–GnP nanocomposites increased at low filler loadings (0.5 and 1.0 wt.%) compared to the neat PEEK, followed by a decline in modulus at higher loading, i.e., at 3.0 wt.% of GnPs. Significant improvement in the storage modulus was observed with the addition of GnPs, revealing strong reinforcing action of the GnP layers by effectively absorbing the load from the PEEK polymer chains. Among all the compositions, the PEEK–GnP1.0 nanocomposite (1.0 wt.% of GnPs) showed the highest improvement ~61% in the storage modulus, at 30 °C concerning neat PEEK, as shown in Figure 8a. In particular, the storage modulus of the sample PEEK–GnP1.0 increased from 2458 ± 88 MPa (for the neat PEEK) to 3958 ± 176 MPa. The increment of 61% in the storage modulus of the sample PEEK–GnP1.0 is the highest improvement reported for PEEK nanocomposites, to the best of our knowledge, as reported in Table 3 (refer to Table 3). The plausible reason for such an improvement in the storage modulus of the PEEK–GnP1.0 nanocomposite could be explained by the improved GnP dispersion and more robust interface of GnP-PEEK via the sophisticated chemical hydrogen bonding and π–π* interactions, which can oppose the PEEK molecular motions at the interfaces, thus improving the interfacial contacts causing greater stress transfer between GnP and PEEK interfaces [20]. Figure 8b shows the tanδ curves of the neat PEEK and PEEK–GnP nanocomposites. Enhancement in glass transition temperature (*T*_g_) was noticed from tanδ curves in the presence of GnPs than the pristine PEEK. It indicates that the unconstrained segments of the PEEK polymer retain the *T*_g_ of bulk polymer, whereas the PEEK segments in the vicinity of GnP fillers are less mobile due to enhanced interfacial interactions (hydrogen bonding and π–π*) leading to an increase in the *T*_g_ with the maximum shift being from 162.5 °C to 168.2 °C at 1.0 wt.% of GnP loading (see Figure 8c).

### 3.5. Theoretical Modeling of the Mechanical Properties of the PEEK–GnP Nanocomposites

Many theoretical models are employed to predict mechanical properties (modulus of elasticity) of the polymer nanocomposites based on the properties of the individual constituent materials (fillers and matrix) [64]. Furthermore, the filler’s geometry (i.e., aspect ratio), the volume fraction of the filler, orientation, and filler–matrix compatibility play an essential role in the polymer composite’s mechanical enhancement. In particular, the aspect ratio and volume fraction of the fillers are key parameters in such models to correlate theoretical predictions with the experimental results [65,66,67].

**Figure 9:**
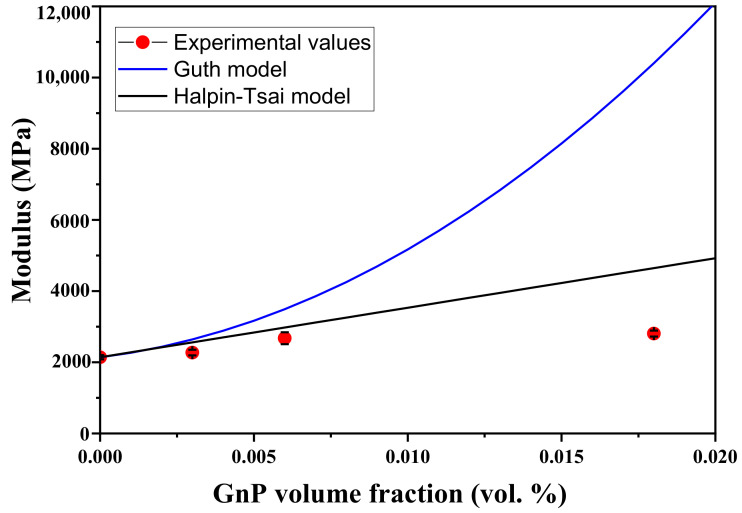
Comparison of experimental modulus with theoretical predictions of nanocomposite models.

Two models from Guth [65] and Halpin–Tsai [66,67] are extensively used for polymer-based composite or nanocomposite materials. The Guth model assumes that an ideal state of the filler–matrix interfacial interaction is present [65]. In comparison, the Halpin–Tsai model is used when fillers’ dispersion is excellent throughout the polymer matrix [66,67]. However, in the real polymer–filler system, these two factors (ideal dispersion and filler–matrix interaction) are difficult to control. Herein, these two models’ theoretical predictions are fitted with the experimental outcomes (modulus values) of the neat PEEK and PEEK–GnP nanocomposites and are plotted in Figure 9. The theoretical details on these models are given in Supplementary Materials, Section SI-5. Both Halpin–Tsai and Guth equations predicted very well the experimental values at lower volume fractions of the fillers (≤0.006 vol%, corresponding to 1.0 wt.%), indicating an improved dispersion of the GnPs inside the PEEK (see SEM images in Supplementary Figure S2b–c) and filler–matrix interaction through H-bonding (see Figure 3). However, the experimental value at higher volume fraction (i.e., 0.018 vol%, corresponding to 3.0 wt.%) deviated from both Halpin–Tsai and Guth equations. In the case of Halpin–Tsai model, a small deviation of the experimental data from the predicted values is observed. It can be associated with the formation of GnP aggregates at a higher concentration of the GnPs, as confirmed from the SEM analysis (see Supplementary Figure S2d).

In contrast, the Guth model displayed a large deviation from the experimental values. This indicated that at a higher GnPs loading (i.e., 3 wt.%), filler–filler interactions [68,69,70] through weak Van der Waals forces dominate concerning the H-bonding and π–π* interactions with the polymer at the interfaces (refer Section 3.2), leading to filler agglomerates. These agglomerates not only create weakly bonded spots within the polymer matrix but also can cause voids upon pulling (see Supplementary Figure S2d). When the nanocomposites are pulled apart, the formed voids reduce matrix–filler stress transfer efficiency and the nanocomposites show a significant reduction in the mechanical performance. Therefore, a slight agglomeration of the fillers causes a huge deviation from the predicted values in the Guth model. In Figure 9, both conditions of the high filler dispersion and filler–matrix interaction are fulfilled at only 0.5 wt.%, and 1.0 wt.% of GnP loading (confirmed with SEM and FTIR analysis) and consequently, the experimental values of Young’s modulus for the PEEK–GnP nanocomposites show perfect match with the predicted ones. To conclude, the PEEK–GnP nanocomposite, optimized at 1.0 wt.% of GnPs loading, has demonstrated uniform surface morphology, good thermal stability and excellent mechanical performance and can be used for structural applications in robotics, transportation, aviation and medical instrumentation through 3D printing or injection molding techniques.

## 4. Conclusions

Nanocomposites of the high-performance thermoplastic PEEK polymer with GnPs were developed by initially dispersing the GnP powder in ethanol by sonication and mixing it with pulverized PEEK particles (size ~1 mm) using a mechanical homogenizer until dry. The GnPs-decorated PEEK powder was subsequently melt-extruded using a twin-screw extruder. The extruded PEEK–GnP nanocomposite filaments with 1.0 wt.% of GnPs (sample PEEK/GnP1.0) demonstrated excellent mechanical properties, with significant improvement in Young’s modulus (25%), tensile strength (34%), elongation at break (31%), and storage modulus (61%) compared to the neat PEEK polymer. In particular, Young’s modulus, tensile strength, elongation at break, and storage modulus increased from ~2002 MPa, ~104 MPa, ~192% and ~2458 MPa (for neat PEEK) to ~2529 MPa, ~139 MPa, ~253%, and ~3958 MPa, respectively, due to the enhanced dispersion of the GnP fillers and formation of strong interfaces via H-bonding and π–π* interactions with the PEEK matrix. On the top, the addition of GnPs to the PEEK matrix enhances its thermal stability. Halpin–Tsai and Guth theoretical models verified the improved dispersion and the optimized reinforcement of GnP inside PEEK, respectively, at lower loadings (0.5 and 1.0 wt.%). The extruded PEEK–GnP nanocomposite can find practical implications in the transportation sector, robotic technology, and other demanding engineering sectors by developing highly resistive structural body parts using scaled-up technologies like injection molding 3D printing.

## Figures and Tables

**Table 1:** Composition of PEEK–GnP nanocomposites.

Sample Name	Polymer Weight (Wt %)	Graphene Weight (Wt %)
Neat PEEK	100.0	0.0
PEEK–GnP0.5	99.5	0.5
PEEK–GnP1.0	99.0	1.0
PEEK–GnP3.0	97.0	3.0

**Table 2:** Degree of crystallization (X_c_) of the NEAT PEEK and PEEK–GnP nanocomposites.

Sample Name	X_c_ (%)
Neat PEEK	29.1
PEEK–GnP0.5	28.5
PEEK–GnP1.0	27.4
PEEK–GnP3.0	26.1

**Table 3:** Literature review of mechanical properties of the previously reported PEEK nanocomposites.

Polymer	Filler Type	Concentration (wt.%)	Improvement in Ultimate Tensile Strength (%)	Improvement in Young’s Modulus (%)	Storage Modulus (%)	Reference
PEEK	GnP	1.0	34	25	61	Present study
PEEK PEEK PEEK	GnP GnP MWCNT	10.0 5.0 2.5	−16 * 4 10	44 23 6	- 25 -	[6] [58] [59]
PEEK	Modified GnP	3.0	10	39	-	[61]
PEEK	bi/tri-layer graphene	5.0	-	-	29	[18]
PEEK	GO	2.0	0	4	-	[20]
PEEK	CNF	10.0	40	25	12	[9]
PEEK	Modified SWCNT	1.0	39	14	38	[36]
PEEK	CNF	15.0	50	40	-	[62]
PEEK	Nano-hydroapatite rods + CNF	32.0	−14 *	67	-	[63]
PEEK	MWCNT	17.0	-	50	-	[13]
PEEK	Modified SWCNT	1.0	-	-	34	[12]
PEEK	Modified GO + modified MWCNT	1.5	-	-	50	[25]

* The negative sign shows a decrease in the mechanical properties.

## Supplementary Materials

A supplementary material document is provided with this manuscript to help understand the findings of the presented research work. The following are available online at https://www.mdpi.com/article/10.3390/polym13091425/s1, Figure S1: Step-by-step fabrication process of the neat PEEK and PEEK–GnP filaments by the melt-extrusion process, Figure S2: Cross-sectional SEM images of (a) Neat PEEK and PEEK loaded with (b) 0.5 wt.% (c) 1.0 wt.% (d) 3.0 wt.% of GnP fillers (red arrows indicate GnP layers and yellow arrows indicate voids), Figure S3: Deconvoluted C1s XPS spectrum of (a) GnP, (b) GnP (1.0 wt.%) reinforced PEEK (PEEK–GnP1.0) composite., Figure S4: DSC curves (melting) of the neat PEEK and sample PEEK–GnP0.5, PEEK–GnP1.0 and PEEK–GnP3.0, Table S1: Crystallite size of the neat PEEK and PEEK/GnP composites. Referencess [34,48,65,66,67,71,72,73,74] are cited in the Supplementary Materials.
