# Supplementary material for: Comprehensive Enhancement in Thermomechanical Performance of Melt-Extruded PEEK Filaments by Graphene Incorporation
Source: Polymers (Basel). 2021 Apr 28;13(9):1425. doi: 10.3390/polym13091425 (PMC8124288; doi:10.3390/polym13091425)
Supplement: Supplementary file 1 [file polymers-13-01425-s001.zip › polymers-1186303-supplementary.pdf]

polymers

MDPI

# Supplementary Material: Comprehensive Enhancement in Thermo-Mechanical Performance of Melt Extruded PEEK Filaments by Graphene Incorporation

**Srinivasarao Yaragalla, Muhammad Zahid, Jaya Kumar Panda, Nikolaos Tsagarakis, Roberto Cingolani, Athanassia Athanassiou**

## 1. Fabrication process

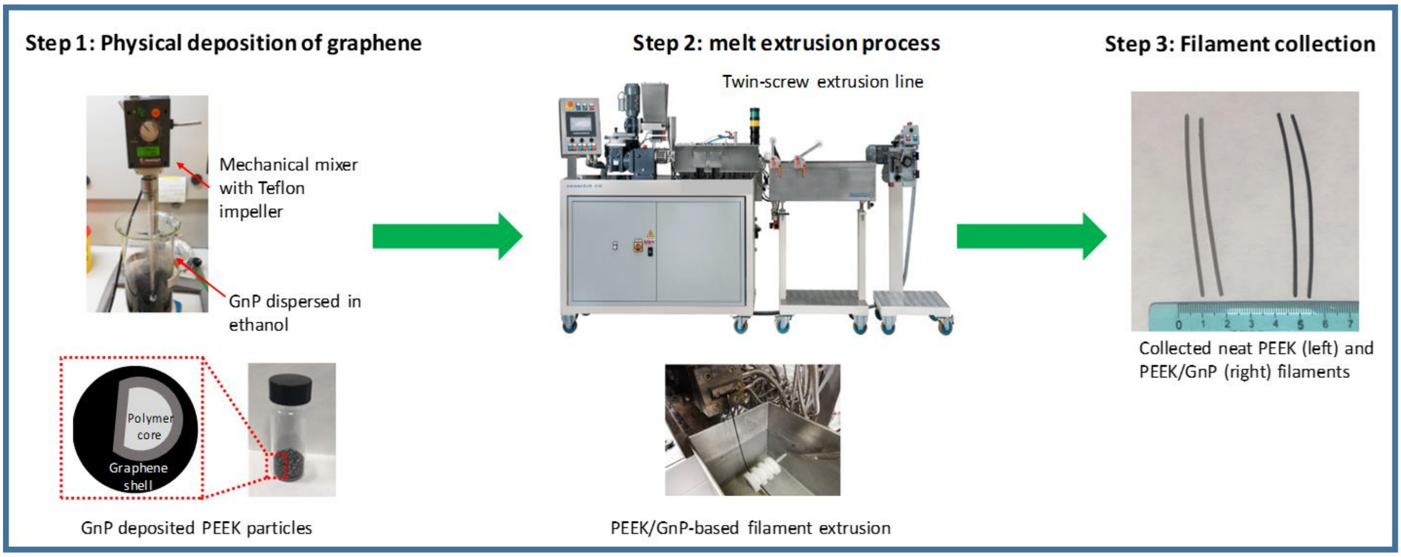


**Figure S1.** Step-by-step fabrication process of the neat PEEK and PEEK-GnP filaments by melt extrusion process.

## 2. Morphology of neat PEEK and PEEK-GnP composites

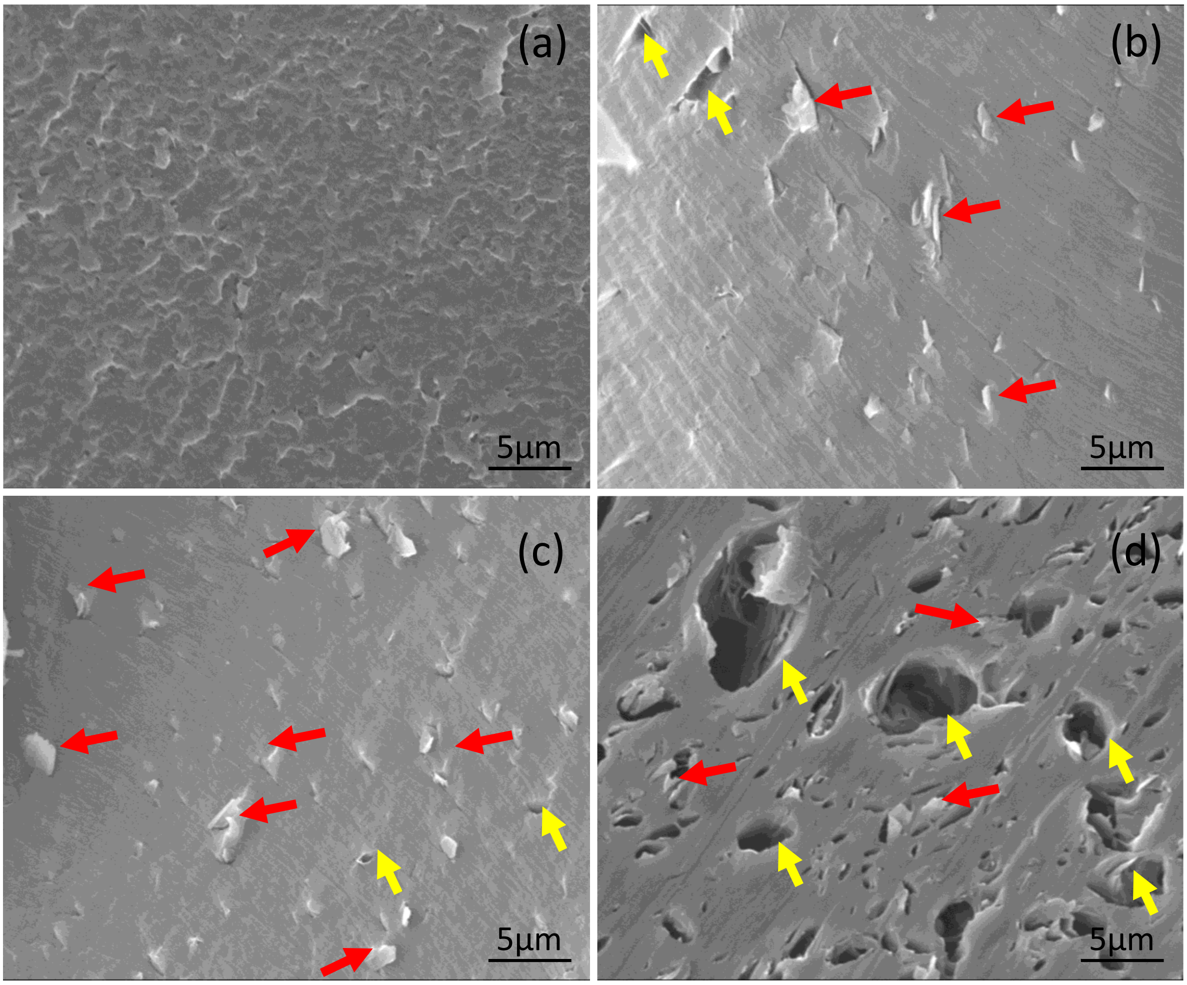


**Figure S2.** Cross-sectional SEM images of (a) Neat PEEK and PEEK loaded with (b) 0.5 wt. % (c) 1.0 wt. % (d) 3.0 wt. % of GnP fillers (red arrows indicate GnP layers and yellow arrows indicate voids).

## 3. X-ray photo-electron spectroscopy (XPS) analysis

**Figure S3a** shows high-resolution C1s spectra of the chemical functionalities present in the GnP powder used herein. The C1s spectra of the GnP was fitted with three different peaks related to various carbon environments. The peak at 284.46 belongs to conjugated aromatic C-C bonds of hexagonal lattice [1] and the other peaks at 286.55 and 290.55 could be assigned to the C-O groups and $\pi$-$\pi^*$ interactions, respectively [2].

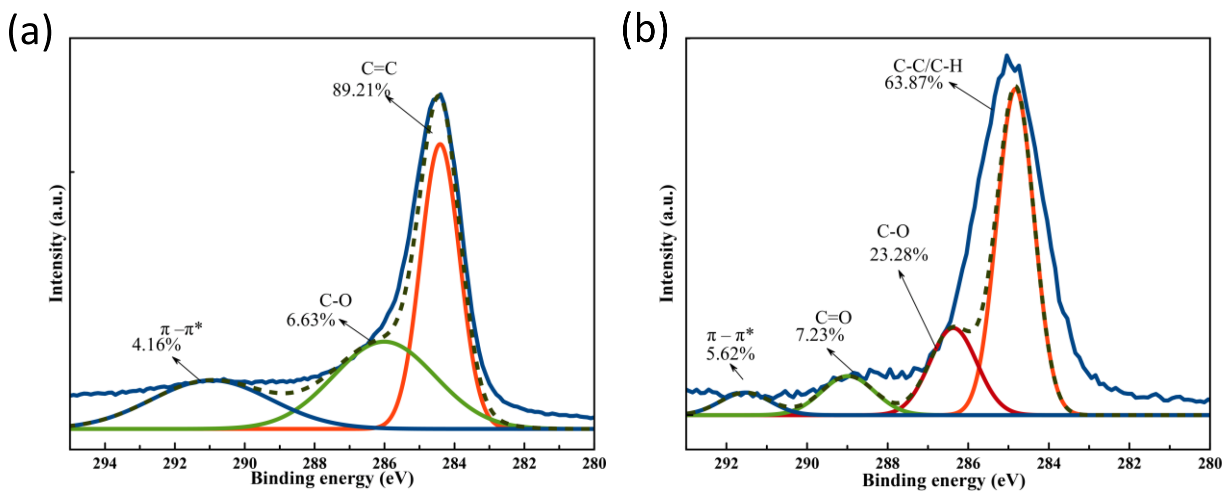


**Figure S3.** Deconvoluted C1s XPS spectrum of (a) GnP and (b) GnP (1.0 wt%) reinforced PEEK (PEEK-GnP1.0) composite.

## 4. Estimation of crystallite size of neat PEEK and PEEK-GnP composites

Crystallite size in the neat PEEK and PEEK-GnP composites was estimated using **Scherrer equation (1)** as follows [3] and listed in the Table S1,

$$D = \frac{K\lambda}{\beta \cos\theta} \quad (1)$$

where $D$ is the crystallite size, $\theta$ represents Brags angle (peak position in radians), $K$ and $\lambda$ indicate Scherrer constant (0.9) [4] and the wavelength of the X-ray (0.15406 nm), respectively. $\beta$ denotes the full width at half maximum (FWHM) of the diffraction peak.

**Table S1.** Crystallite size of the neat PEEK and PEEK/GnP composites.

| Sample name | Crystallite size (nm) |
|---|---|
| Neat PEEK | 6.43 |
| PEEK-GnP0.5 | 6.22 |
| PEEK-GnP1.0 | 6.17 |
| PEEK-GnP3.0 | 5.63 |

*4.1. DSC analysis of PEEK-GnP composites*

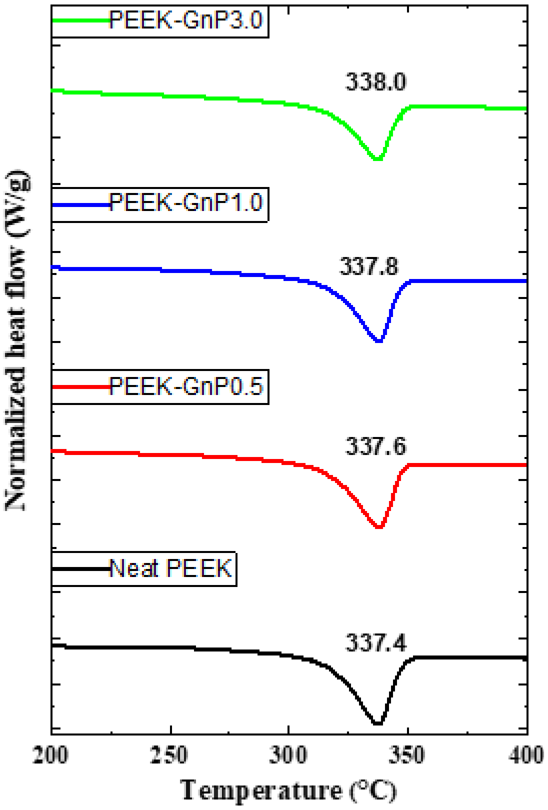


**Figure S4. Second heating** DSC curves (melting) of the neat PEEK and sample PEEK-GnP0.5, PEEK-GnP1.0 and PEEK-GnP3.0.

## 5. Theoretical composite models

The Guth equation [5] mainly considers the hydrodynamic effect of rigid nanofillers inside polymers and is derived based on the findings of Smallwood who modified the Einstein equation by introducing a quadratic term to estimate the interactions associated with filler particles. In continuation to this, Guth postulated that the effect of reinforcement of the fillers inside polymer is enhanced by their strong interfaces, based on this, he derived an equation, which is in relation with volume fraction of the filler and the elastic modulus of the polymer.Øη

$$E = E_m(1+0.67A_f Ø_f + 1.62(A_f Ø_f)^2) \quad (2)$$

where $E$ is the transverse composite modulus, $E_m$ represent the matrix modulus (2142 MPa), $A_f$ and $Ø_f$ are related to aspect ratio and volume fraction of the filler (converted from weight percent based on density of graphene 2.28 g/cm$^3$), respectively. While aspect ratio was taken as 75 from our previous research [6].

The Halpin-Tsai model [7, 8] is a semi empirical model which, considers that fillers are distributed homogeneously inside the polymer matrix and the equation is as follows,

$$E = E_m(1+\varepsilon\eta Ø_f)/(1-\eta Ø_f) \quad (3)$$

$$\text{where} \quad \eta = (\frac{E_f}{E_m}) - 1/(\frac{E_f}{E_m}) + \varepsilon$$

where $E_m$ and $E_f$ are modulus of polymer and filler respectively, $A_f$ is the aspect ratio, ε is shape factor and $Ø_f$ is the volume fraction of the filler. A shape factor of $\varepsilon=2A_f$ is used for

the fillers that are oriented with the loading direction [7]. Modulus of graphene is taken as 250 GPa [9] for the purpose of this analysis.

## References

[1] Yaragalla, S.; Rajendran, R.; Jose, J.; Almaadeed, M.A.; Kalarikkal, N.; Thomas, S. Preparation and characterization of green graphene using grape seed extract for bioapplications. *Mater Sci Eng C* **2016**, doi: 10.1016/j.msec.2016.04.050.
[2] Dave, K.; Park, K.H.; Dhayal M. Characteristics of ultrasonication assisted assembly of gold nanoparticles in hydrazine reduced graphene oxide. *RSC Adv* **2015**, *5*, 107348–54, doi:10.1039/C5RA23018G.
[3] Muniz FTL, Miranda MAR, Morilla Dos Santos C, Sasaki JM. The Scherrer equation and the dynamical theory of X-ray diffraction. *Acta Crystallogr Sect A Found Adv* **2016**, doi:10.1107/S205327331600365X.
[4] James, W. *The Optical Principles of the Diffraction of X-rays. In The Crystalline State*; Bragg, W. L., Eds.; G. Bell and Sons Ltd.: London, UK, 1962, Vol. II.
[5] Wu, Y.-P.; Jia, Q.-X.; Yu, D.-S.; Zhang, L.-Q. Modeling Young’s modulus of rubber–clay nanocomposites using composite theories. *Polym. Test* **2004**, *23*, 903–909.
[6] Cataldi, P.; Bayer, I.S.; Nanni, G.; Athanassiou, A.; Bonaccorso, F.; Pellegrini, V.; del Rio Castillo A.E., Ricciardella, F.; Artyukhin, S.; Tronche, M. A.; Gogotsi, Y.; Cingolani, R.. Effect of Graphene Nano-Platelet Morphology on the Elastic Modulus of Soft and Hard Biopolymers. *Carbon* **2016**, *109*, 331–339, doi:10.1016/j.carbon.2016.08.026.
[7] Halpin, J.C.; Kardos, J.L. The Halpin-Tsai Equations: A Review. *Polym. Eng. Sci.* **1976**, *16*, 344–352, doi:10.1002/pen.760160512.
[8] Mittal, V. Modeling of Tensile Modulus of Polyolefin-Layered Silicate Nanocomposites: Modified Halpin Tsai Models. *Adv. Compos. Lett.* **2012**, doi:10.1177/096369351202100501.
[9] Gómez-Navarro, C.; Burghard M.; Kern, K. Elastic Properties of Chemically Derived Single Graphene Sheets. *Nano Lett* **2008**, *8*, 2045–2049, doi:10.1021/nl801384y.
